# Effects of apolipoprotein E isoform, sex, and diet on insulin BBB pharmacokinetics in mice

**DOI:** 10.1038/s41598-021-98061-1

**Published:** 2021-09-20

**Authors:** Elizabeth M. Rhea, Kim Hansen, Sarah Pemberton, Eileen Ruth S. Torres, Sarah Holden, Jacob Raber, William A. Banks

**Affiliations:** 1 Department of Medicine, Division of Gerontology and Geriatric Medicine, University of Washington, Seattle, WA 98195 USA; 2 Geriatric Research Education and Clinical Center, Veterans Affairs Puget Sound Health Care System, Seattle, WA 98108 USA; 3 Department of Behavioral Neuroscience, Oregon Health & Science University, Portland, OR 97239 USA; 4 Division of Neuroscience, Departments of Neurology and Radiation Medicine, ONPRC, Oregon Health & Science University, Portland, OR 97239 USA

**Keywords:** Neuroscience, Physiology

## Abstract

Age, apolipoprotein E (apoE) isoform, sex, and diet can independently affect the risk for the development of Alzheimer’s disease (AD). Additionally, synergy between some of these risk factors have been observed. However, the relation between the latter three risk factors has not been investigated. Central nervous system (CNS) insulin resistance is commonly involved in each of these risk factors. CNS insulin is primarily derived from the periphery in which insulin must be transported across the blood–brain barrier (BBB). Additionally, insulin can bind the brain endothelial cell to affect intracellular signaling. Therefore, we hypothesized CNS access to insulin could be affected by the combination of apoE isoform, sex, and diet. We analyzed insulin BBB pharmacokinetics in aged apoE targeted replacement (E3 and E4) male and female mice on a low-fat and high-fat diet. There were differences within males and females due to apoE genotype and diet in insulin interactions at the BBB. These sex-, diet-, and apoE isoform-dependent differences could contribute to the cognitive changes observed due to altered CNS insulin signaling.

## Introduction

Impairments in the regulation of central nervous system (CNS) insulin are associated not only with metabolic syndrome and diabetes mellitus but also with Alzheimer’s disease (AD), age-related cognitive decline, and mild cognitive impairment (MCI). MCI and AD-related dementia (ADRD) is an epidemic that is growing rapidly. Although age is the greatest risk factor for developing age-related cognitive decline, there are many other risk factors involved that can accelerate this decline, including apolipoprotein E4 (apoE4 or E4), female sex, and high-fat diet (HFD). However, it remains unclear how these factors affect age-related cognitive decline. The common thread between these risk factors is insulin resistance in the CNS, defined broadly in this context as impaired insulin activity in the CNS. Indeed, not only is a deficiency in CNS insulin action associated with cognitive impairments, but these risk factors are associated with an impaired cognitive response to insulin delivered therapeutically to the CNS by the intranasal route.

CNS insulin activity is impaired in AD^1^. Cerebrospinal fluid (CSF) to plasma ratios for insulin are lower in patients with AD patients compared to healthy age-matched controls and is more evident in E4 carriers^2,3^. Insulin signaling proteins are decreased in the brains of AD patients^4–7^. In addition, there is an impaired response to ex vivo insulin stimulation in the cerebral cortex and hippocampal formation of post-mortem AD brains compared to healthy controls^8^. This study was critical to show a functional impairment in response to insulin in samples from people with AD. These results point to a disruption in brain insulin metabolism in AD.

Insulin in the CNS is primarily derived from blood insulin, which has been transported across the blood–brain barrier (BBB)^9–11^. Insulin transport into the brain is decreased with obesity and peripheral insulin resistance^12,13^. In addition, CSF insulin levels are decreased in obese animals and humans and correlate with peripheral insulin sensitivity, creating a link between obesity and central insulin dysregulation^12–15^. Obesity and a HFD can impair cognition, especially in women^16,17^. Rodents fed a HFD show cognitive impairments^18–20^ as well as neuronal insulin resistance^21^.

The E4 isoform increases the risk for developing AD^22–25^ and genome-wide association studies confirm that E4 is the most potent genetic risk factor for developing AD^26,27^. In addition, people with diabetes carrying E4 are more predisposed to developing AD than those without E4^28,29^. These data highlight the complex connection between insulin signaling, apoE isoform, and AD risk (for a recent review, see Ref.^30^).

Lastly, female sex is a risk factor for developing MCI and AD^31,32^ and thus is a critical biological variable to consider. The E4 isoform causes an increased risk of developing MCI and AD in females, and female E4 carriers without AD decline more quickly compared to male E4 carriers without AD^33,34^. Consistent with the human findings, female mice expressing E4 in the brain are more susceptible to cognitive impairments than female mice expressing E3 or male mice expressing E4^35,36^. In addition, mice expressing E4 in brain are more severely impaired in an age- and sex-dependent manner in spatial learning and memory compared to mice expressing E3 in brain^35,37,38^. In E4 mice, these impairments occur by 6 months of age in female mice, yet by 18 months of age, male mice are still cognitively intact^37^. Intranasal insulin is a therapy currently under investigation to improve memory in AD. However, men and women non-E4 carriers respond differently than E4-carriers^39^.

**Figure 1:**
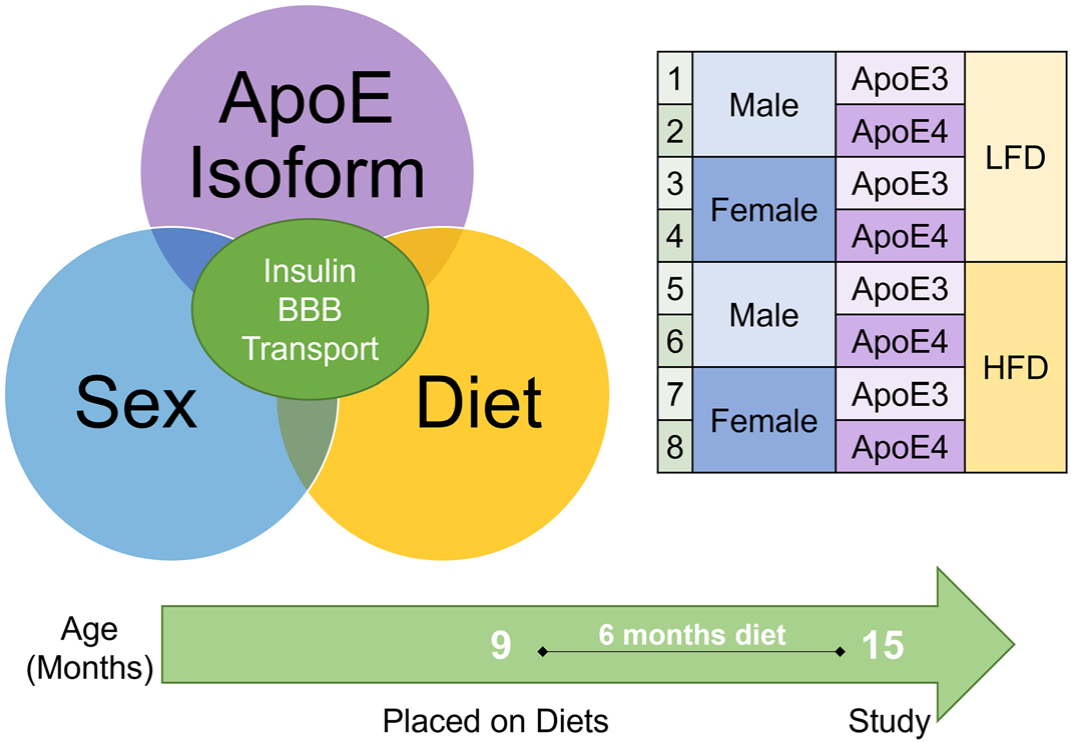
Schematic of the experimental design and interaction between apoE isoform, sex, and diet on insulin BBB pharmacokinetics in aged mice. Groups investigated are listed on the right side and the timeline of the study design presented on the bottom.

We recently investigated the transport of insulin across the BBB in young male and female E3 and E4 mice in order to determine if these factors predisposed young mice to changes in insulin BBB pharmacokinetics^40^. While there were no differences in the transport rate, there were regional differences in the level of reversible insulin binding, suggesting early differences in the interactions of insulin at the BBB. However, we were unable to investigate the impact of age or diet in combination with sex and apoE isoform in this young mouse study. The purpose and novelty of the current study was to define the role of apoE isoform, sex, and diet on insulin BBB pharmacokinetics in older mice as a means for explaining some of the interactions between CNS insulin signaling and these factors (Fig. 1).

## Results

### Body weight

**Figure 2:**
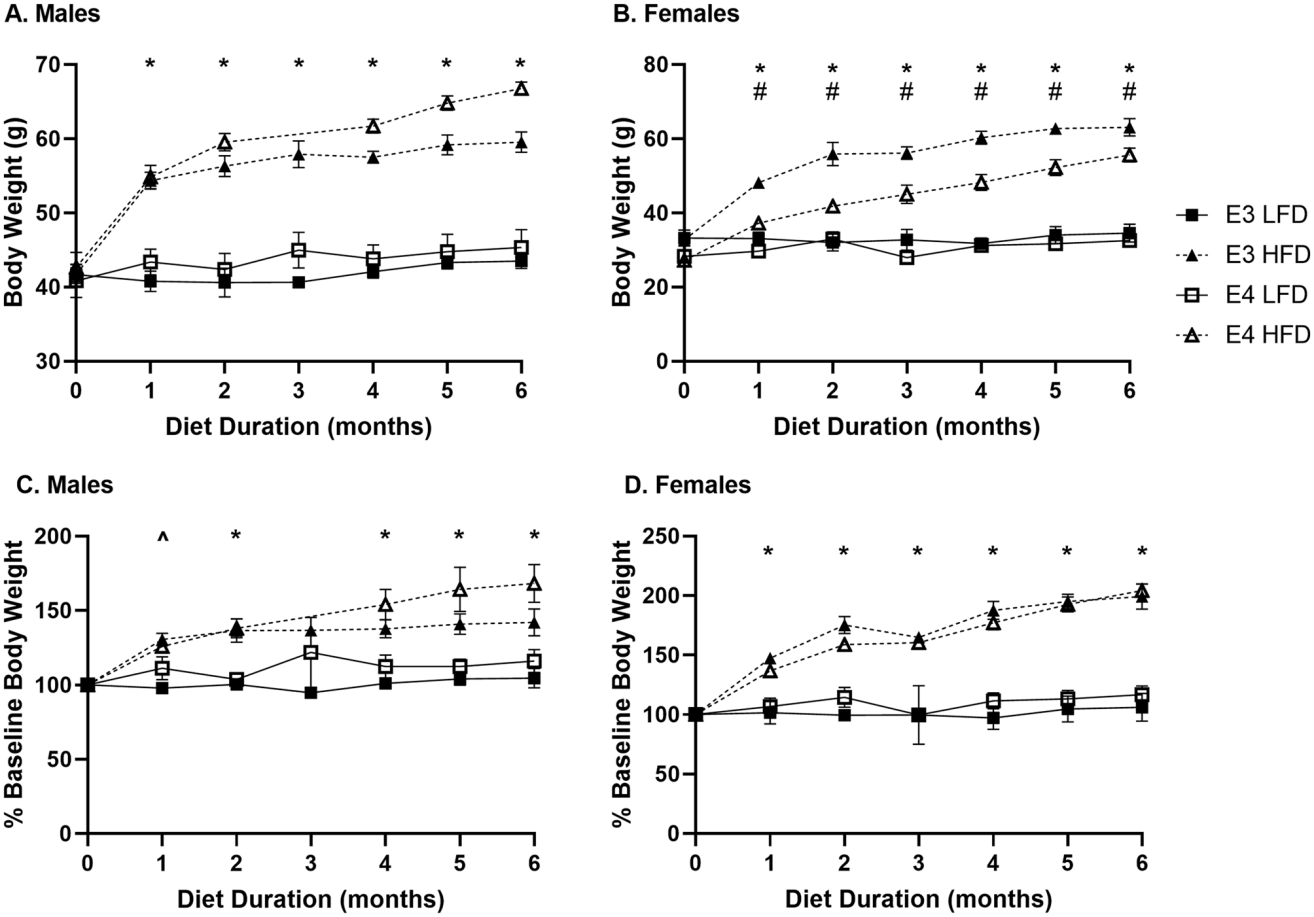
Body weight curves. (**A**) Male raw body weight (g) increases over 6 months of HFD compared to LFD. (**B**) E3 females on a HFD gain more body weight than E4 females on a HFD. (**C**) Male and (**D**) female body weights relative to the starting body weight are increased compared to baseline on due to a HFD. Two-way ANOVA: *p < 0.05 vs respective LFD group. ^#^*p* < 0.05 vs genotype within diet. ^*p* < 0.05 for only E3 HFD vs E3 LFD. n = Males: E3 LFD = 10, E3 HFD = 4–9, E4 LFD = 10, E4 HFD = 7–10; Females: E3 LFD = 9–10; all other female groups n = 10.

Male and female mice on a HFD gained weight over the course of the 6-months as expected (Fig. 2). There were genotype differences in body weight for females on a HFD (Fig. 2B). E3 female mice on a HFD gained more weight than E4 HFD female mice over the 6 months (Fig. 2B, Supplementary Fig. 1).

### Brain region weights

While there was no difference in whole brain weights of the 8 mouse groups, there were differences amongst the groups for regional brain weights (Supplementary Fig. 2). For males and females, there was a significant interaction between brain region weights and genotype (Males: *F*(12, 408) = 2.601, *p* = 0.002; Females: *F*(12, 420) = 2.101, *p* = 0.016). In males, there was no significant effect of diet. However, in E3 females there was a significant effect of diet (*F*(12, 204) = 2.228, *p* = 0.012). In E4 females, there was no significant effect of diet.

### Vascular space

**Figure 3:**
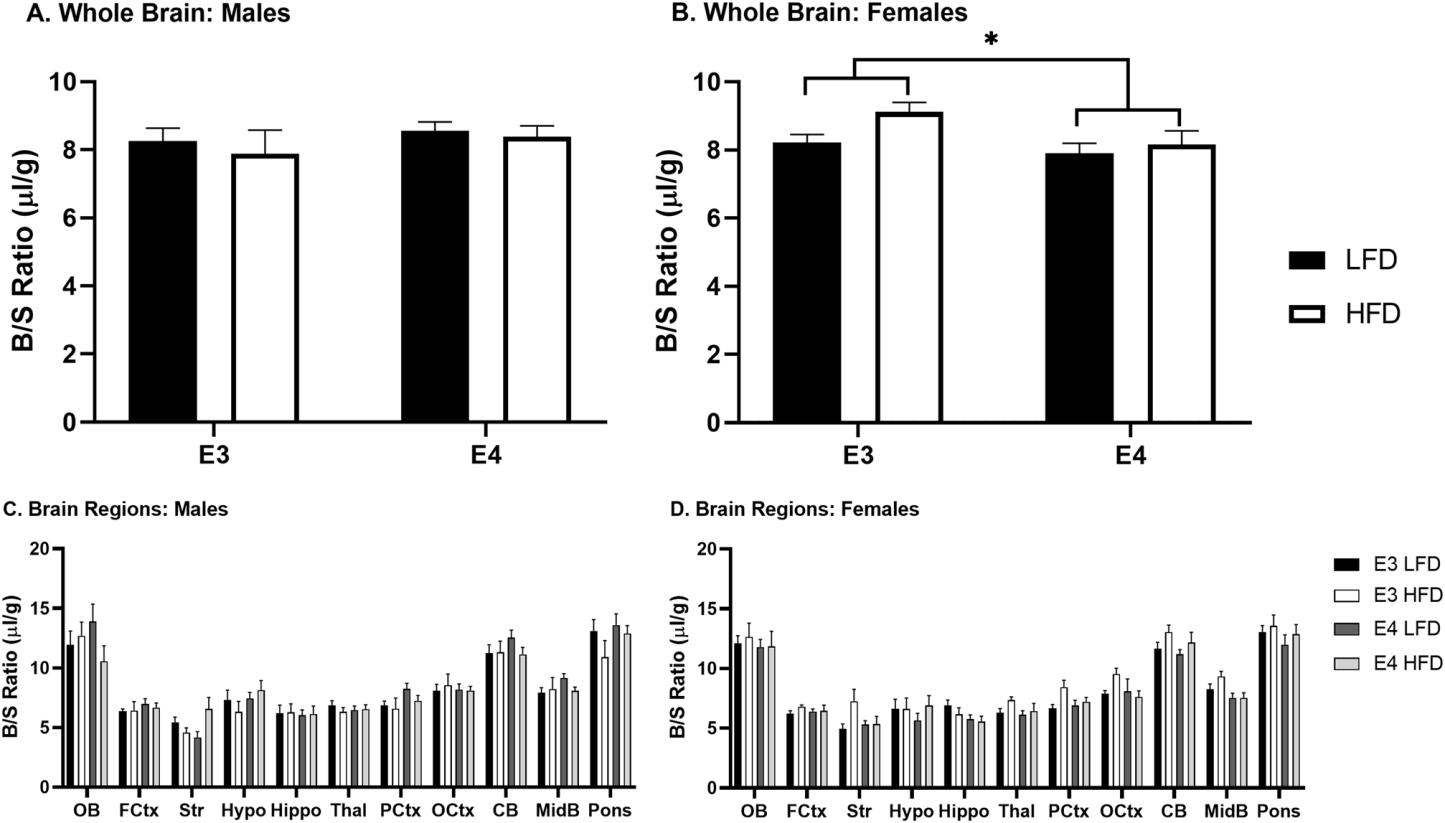
Vascular space. Whole brain vascular space for (**A**) males and (**B**) females were analyzed by a two-way ANOVA and females had significant differences due to genotype (*p < 0.05). Brain region vascular space for (**C**) males and (**D**) females were analyzed by repeated measures. Males had a significant interaction between vascular space, diet, and genotype (*p* < 0.05). There were no brain region differences for females. There were not post-hoc differences. n = Males: E3 LFD = 14, E3 HFD = 7, E4 LFD = 9, E4 HFD = 7; Females: E3 LFD = 9, E3 HFD = 10, E4 LFD = 10, E4 HFD = 9.

As described in methods, mice received an intravenous injection of the vascular marker ^99m^Tc-albumin. Changes in ^99m^Tc-albumin over time would be consistent with BBB disruption. There was no change in ^99m^Tc-albumin levels over time in whole brain (data not shown), demonstrating that there was no BBB disruption as measured by this method. Therefore, ^99m^Tc-albumin can be used to measure the vascular space of the brain. Timepoints were combined to increase power in the assessment of vascular space. There were genotype differences in the vascular space for whole brain for females (Fig. 3, *F*(1, 34) = 4.328, *p* = 0.045). Between brain regions, for males, there was a significant interaction between vascular measures, diet, and genotype (*F*(11, 363) = 2.039, *p* = 0.024). In E3 males, there were no significant effects of diet. However, in E4 males there was a significant effect of diet (*F*(11, 154) = 2.331, *p* = 0.011). For females, there were no significant within-subjects’ effects. There was a trend toward genotype differences (*F*(1, 34) = 4.134, *p* = 0.050) but it did not reach significance. In E3 females, there was a trend toward an effect of diet (*F*(1, 17) = 4.419, *p* = 0.051). In contrast, in E4 females, there were no significant effects of diet.

### Insulin BBB pharmacokinetics

**Figure 4:**
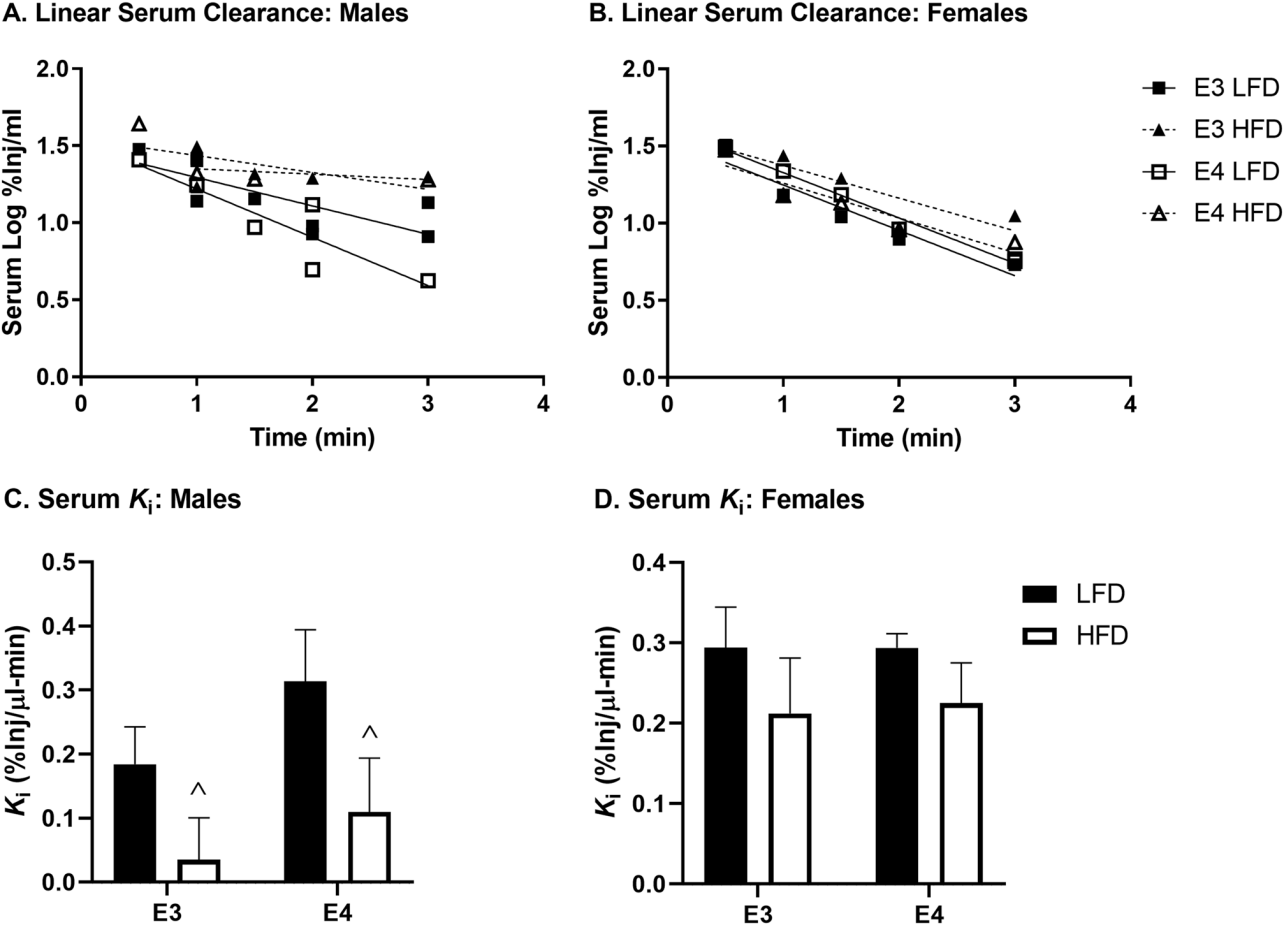
Linear ^125^I-insulin serum clearance. ^125^I-insulin serum clearance curves for (**A**) males were decreased due to HFD compared to LFD but did not differ in (**B**) females. Clearance rates (*K*_i_) were calculated for (**C**) males and were significantly different due to diet (^*p* < 0.05) but not for (**D**) females. There were not post-hoc differences.

The clearance rate from blood of insulin is an important component of insulin pharmacokinetics. As assessment of BBB transport of insulin requires us to inject ^125^I-insulin into the blood and to take blood samples, we are able to assess clearance of insulin by this method. Serum clearance of ^125^I-insulin was significantly slower in males on a HFD than a LFD (Fig. 4A/C, *F*(1, 20) = 5.599, *p* = 0.028). There were no differences due to genotype or diet in females (Fig. 4B/D). Entire serum clearance curves are presented in Supplementary Fig. 3.

**Figure 5:**
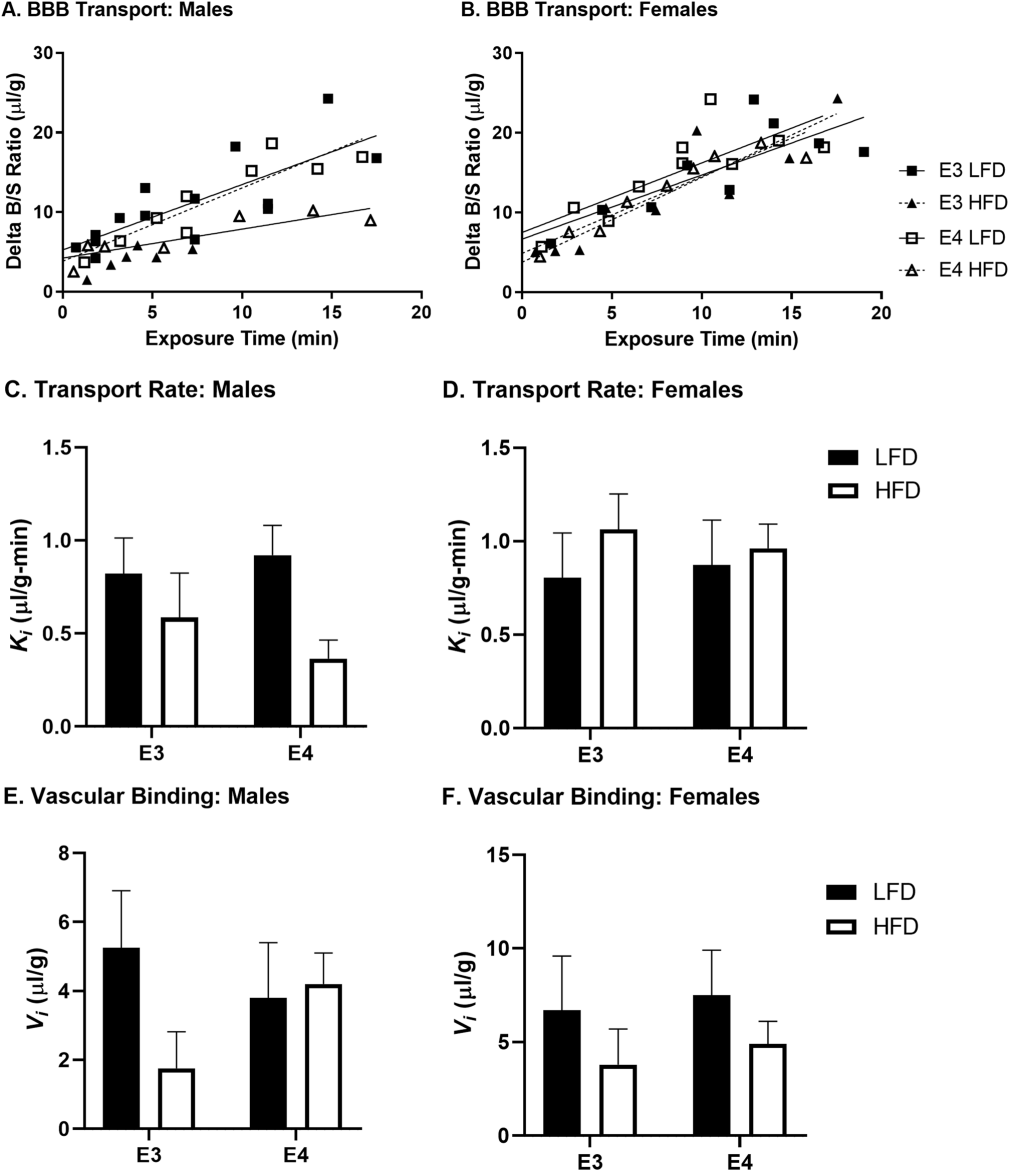
^125^I-insulin BBB transport. Delta B/S values are plotted against exposure time for (**A**) males and (**B**) females. ^125^I-insulin BBB transport rates (*K*_i_) are calculated from the curves in A/B for (**C**) males and (**D**) females. Males have decreases (*p* = 0.056) in BBB transport due to HFD while females do not. The level of ^125^I-insulin vascular binding (*V*_i_) was affected by apoE isoform and diet in (**E**) males (*p* < 0.01) but not in (**F**) females. There were not post-hoc differences.

We next assessed the rate of transport (Ki, or unidirectional influx rate) of ^125^I-insulin across the BBB. Despite decreases in the whole brain BBB transport rate for ^125^I-insulin in males due to HFD, this did not reach significance (Fig. 5A/C, *p* = 0.056). This is likely due to the relatively small n as previous studies have shown decreases ^125^I-insulin BBB transport in obese male mice^41^. We also assessed the reversible binding, or Vi, which measures receptor binding to the brain endothelial cell and vascular space. As we subtracted vascular space as measured with ^99m^Tc-albumin, the Vi here reflected mainly receptor binding. There were differences in the level of whole brain reversible vascular binding (y-axis intercept) for ^125^I-insulin in males (Fig. 5A/E, *p* = 0.002), suggesting alterations in the ability of ^125^I-insulin to interact with the luminal surface of brain endothelial cells. Regional differences in ^125^I-insulin vascular binding were observed in the cortex, hippocampus, cerebellum, and midbrain in males (Table 1). Decreases in binding were predominantly driven by the HFD. There were no differences in whole brain ^125^I-insulin BBB transport or vascular binding in females due to genotype or diet (Fig. 5B/D/F; Supplementary Table 1).

**Table 1:** ^125^I-insulin regional vascular binding in males.

MALES	E3 LFD		E3 HFD		E4 LFD		E4 HFD		*p*
Region	*V*_i_	SEM	*V*_i_	SEM	*V*_i_	SEM	*V*_i_	SEM	
Olfactory bulb	5.9	3.3	0.4	2.0	4.8	3.5	4.8	2.4	
Frontal cortex*	**4.0**	**1.1**	**2.5**	**1.4**	**3.8**	**1.5**	**3.9**	**0.7**	**0.008**
Striatum	3.6	2.5	2.5	1.6	4.4	3.2	4.2	3.2	
Hypothalamus	3.8	3.5	1.9	3.3	8.0	2.8	3.2	3.1	
Hippocampus*	**7.1**	**3.2**	**1.5**	**3.6**	**4.7**	**4.4**	**3.0**	**1.6**	**0.026**
Thalamus	6.7	2.3	2.3	1.1	4.1	3.8	6.2	1.4	
Parietal cortex*	**5.8**	**1.5**	**3.8**	**1.3**	**3.4**	**2.6**	**2.7**	**1.1**	**0.006**
Occipital cortex*	**5.0**	**2.0**	**0.2**	**1.4**	**4.1**	**2.1**	**3.3**	**1.4**	**0.017**
Cerebellum*	**7.5**	**2.5**	**1.0**	**1.5**	**8.1**	**3.0**	**6.3**	**1.3**	**0.0003**
Midbrain*	**4.5**	**1.3**	**− 1.1**	**1.9**	**3.7**	**1.7**	**3.5**	**0.5**	**0.023**
Pons/medulla	4.5	3.5	1.7	1.2	3.6	3.8	3.4	1.4	

Bold cells represent regions where *p < 0.05 between groups. *V*_i_: reversible vascular binding, SEM: ± standard error mean, *p:* significance between groups.

**Figure 6:**
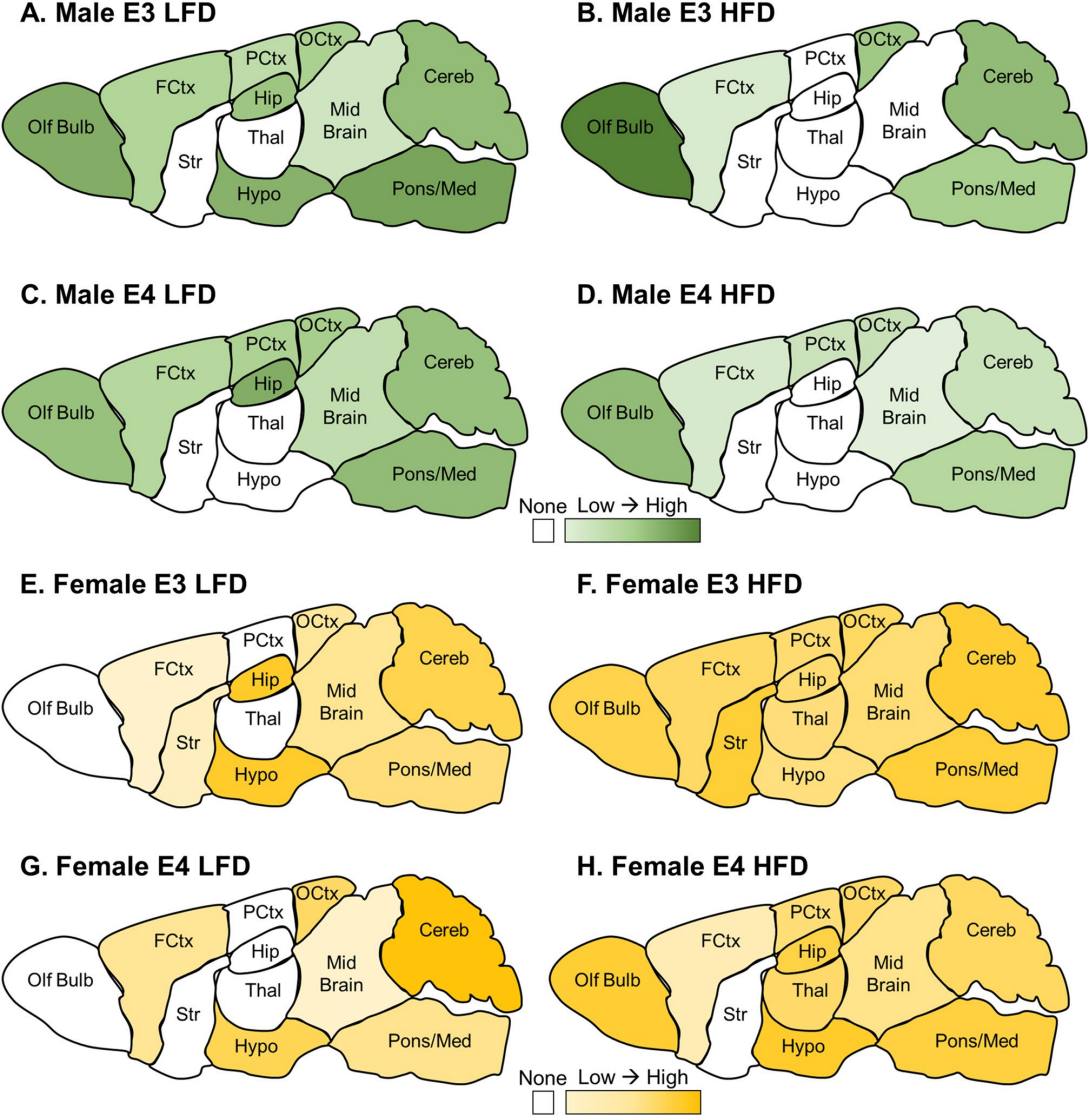
Regional Transport Rates Heat Map (µl/g-min). Heat maps were generated based on the data in Supplementary Table 2 and 3. (**A**–**D**) Males and (**E**,**F**) Females were analyzed separately. The *K*_i_ for each group, within each sex, was color coded based on the rate. White indicates non-significant transport. In males, the olfactory bulb in E3 mice on a HFD had the highest rate of transport whereas in females, the cerebellum in E4 mice on a LFD had the highest rate of transport.

We next assessed the rate of transport of ^125^I-insulin across the BBB of the 11 brain regions, There were a few regions where ^125^I-insulin BBB transport did not occur. In males, the striatum and thalamus did not show significant ^125^I-insulin transport, meaning there was not a statistically significant relation between the brain/serum (B/S) ratio and exposure time (Supplementary Table 2). The hypothalamus and hippocampus also did not exhibit ^125^I-insulin transport in males on a HFD, regardless of genotype. For females, the olfactory bulb, thalamus, and parietal cortex did not show significant transport in the LFD groups (Supplementary Table 3). The striatum did not show transport in E4 mice but did in E3 mice. In the hippocampus, while E3 LFD mice did show ^125^I-insulin transport, this was not the case for E4 LFD mice. These data suggest a HFD in males may impair ^125^I-insulin transport whereas in females it may be beneficial and apoE genotype may be a greater contributing factor in females compared to males. A heat map showing the transport rates in each brain region for each group are presented in Fig. 6. Regions that did not have significant transport are illustrated in white.

## Discussion

In this analysis of insulin pharmacokinetics at the BBB in aged mice due to sex, apoE isoform, and diet, we found marked differences in the cerebrovascular interactions of insulin associated with these factors. First, we found genotype differences between the sexes in body weight gain due to diet and genotype differences in brain weights. We also observed vascular space apoE isoform-dependent differences throughout the brain in both sexes. Serum clearance of insulin was only impacted in males due to diet. However, regional insulin BBB transport was more impacted by diet in females. Lastly, cerebrovascular binding of insulin was more affected by apoE isoform and diet in males.

In the current study, we explored the differences in body weight gain in aged E3 and E4 male and female mice on a LFD and HFD. In previous studies, changes in body weight were analyzed in young apoE targeted replacement female and male mice on a HFD over time. Jones et al. compared metabolic differences in male and female E3 and E4 mice when placed on a 3-month diet at 6-months of age^42^. The HFD used in this study was 45% kcal from fat while the HFD in our study was a 60% diet. Mice gained 30–45% of their starting body weight, with no genotype differences in either sex. We saw similar effects in our study with a longer duration of diet started at an older age when comparing body weights as percent of baseline in individual mice. However, when the absolute body weights were compared, while there were no differences in starting body weight between the groups, female E4 mice on a HFD gained less weight than female E3 mice on a HFD. The data of the current study are consistent with an earlier study by our group in which the effect of a HFD due to apoE genotype was only examined in females^43^. The diets and timeline matched that of the current study. E4 females on a HFD gained less absolute weight than E3 mice on a HFD, similar to the current study. Thus, female mice may be more affected by a HFD due to apoE genotype compared to males. Interactions between obesity and apoE genotype have been reviewed elsewhere^44,45^ and suggest differences in weight gain as a consequence of storage of lipids in adipose tissue could be a contributor to insulin resistance and glucose intolerance.

There were no differences in whole brain weights in males or females. However, there were brain region differences, primarily due to apoE genotype interactions rather than diet. It should be noted, there were significant differences due to diet in E3 female mice but not in E4 female mice. Clinical differences in cortical atrophy due to apoE genotype in patients with mild AD have been reported^46^ and our data further suggests differences in response to diet due to apoE genotype.

Additionally, we measured cerebrovascular space in each group based on our vascular marker, ^99m^Tc-albumin. There were significant decreases in whole brain cerebrovascular space in E4 females independent of diet, with regional differences in both males and females. In our previous study investigating female mice only on the same diet regimen, we found decreases in cerebral blood volume as a measure of vascular perfusion due to E4^43^. In our current study, we further investigated sex differences and regional effects due to apoE genotype and diet on cerebrovascular space. In males, there was a significant interaction between vascular space, diet, and genotype, suggesting a relationship between these 3 factors. In E4 males, there were also significant effects due to diet, while there were no effects of diet in E3 males. The opposite was observed in females. In E3 females, there were significant effects due to diet, while in E4 females there were no diet effects. These sex differences due to diet within particular apoE genotypes highlight the differential effects of diet in males and females on cerebrovascular space due to apoE genotype. Differences in cerebrovascular space could ultimately affect BBB structure and function. Indeed, there is an association between altered cerebrovasculature and neurodegenerative pathology^47^ and there is a building amount of literature between alterations in the BBB and aging or AD^48–50^.

Next, we explored insulin pharmacokinetics in each of our mouse groups. We started by analyzing the serum clearance of insulin. Males on a HFD had a significantly decreased serum clearance rate compared to males on a LFD, without an effect of genotype. This diet effect on serum clearance is consistent with that reported in obese CD-1 male mice^41^. However, females had no effect on insulin serum clearance due to apoE genotype or diet. These sex differences could be due to the metabolic differences discussed above. Indeed, obese men carrying E4 have higher levels of insulin compared to obese men not carrying E4. In women, there was no effect of apoE genotype and obesity on fasting insulin^51^. Further investigations as to why there are sex differences in serum clearance of insulin in obese individuals, potentially dependent on apoE status, are warranted.

The main focus of our study was to investigate the transport of insulin across the BBB amongst our different groups. There were similar, although not statistically significant, decreases in whole brain insulin BBB transport in males on a HFD as previously reported^41^. There was no effect due to apoE genotype in whole brain, nor any differences in females due to apoE genotype or diet. However, there were general differences in regional insulin BBB transport between males and females due to diet and genotype. There were a few regions, primarily in males, in which there was no statistically significant transport of insulin. These regions included the thalamus and striatum. In E3 and E4 males on a HFD there was also no transport into the hippocampus. In females, transport of insulin across the BBB into the olfactory bulb and hypothalamus were affected, primarily due to diet. In the olfactory bulb, thalamus, and parietal cortex insulin BBB transport was enhanced in females on a HFD. In the striatum, E4 female mice did not exhibit insulin BBB transport compared to E3 female mice. In the hypothalamus, female E3 mice on a HFD showed decreased transport while female E4 mice on a HFD showed increased transport. While these findings did not reach significance, a greater sample size may help reduce the variability with the small brain region sampling. In a previous study, in which the regional BBB transport of insulin was investigated in young E3 and E4 male and female mice, significant transport also did not occur in the striatum^40^. The differences in insulin transport between the various brain regions is interesting and the reason is largely unknown but could be due to differences in regional-specificity regulation of the insulin transporter.

In addition to transport kinetics across the BBB, we also explored the level of reversible insulin binding to the cerebrovasculature. In males, the whole brain insulin binding was different between the groups. In E3 males, binding was lower on a HFD than a LFD. Additionally, regions including the cortices, hippocampus, cerebellum, and midbrain also showed decreases in the level of reversible vascular binding due to HFD in males only. In females, there were no differences in the level of vascular binding due to apoE genotype or diet. These data suggest that there is a greater effect of diet on insulin cerebrovascular binding in males than females. In a study comparing young and aged male mice of an AD model, the SAMP8 mice, levels of insulin cerebrovascular binding were increased in the aged mice in the parietal cortex and cerebellum^52^, two regions that showed differences in binding in our study in males. We previously investigated whether apoE genotype and/or sex pre-disposed young mice to changes in insulin interactions at the BBB^40^. Young E4 mice often had increased insulin vascular binding compared to young E3 mice, which is what we observed in aged female mice, with sex differences mostly appearing regionally. Changes in insulin cerebrovascular binding could alter the intracellular signaling within the brain endothelial cell or suggest differences in the level of regional insulin binding sites at the BBB due to apoE genotype, sex, and/or diet.

One major limitation to our study was the number of mice lost in selective groups. The HFD provided had a greater impact on male mortality than female mortality and on mortality of E3 males than E4 males. This was unexpected as previous studies involving only female mice on this same diet regimen displayed no animal loss^43^. The cause of increased mortality in males is unknown. The observed survivorship bias could have contributed to the results seen in male E3 and E4 mice on a HFD. Regardless, we did observe similar decreases in the insulin BBB transport rate in obese E3 and E4 male mice on a C57BL/6J background as previously shown in CD-1 wild-type mice^41^. These data suggest males might be more susceptible for the detrimental effects of a HFD.

In summary, in the present study there were differential effects in male and female mice due to apoE genotype and diet in the interactions of insulin at the BBB. These data could explain differences observed in these groups with regards to CNS insulin signaling, insulin resistance, and response to exogenous insulin therapy. Further mechanistic studies are needed to determine how these interactions at the BBB translate to signaling changes within the CNS. However, the data gained in this pre-clinical study could guide therapeutic strategies when considering delivering insulin to males and females with varying apoE genotypes and body weights.

## Methods

### Animals

Female and male homozygous human E3- and E4-targeted replacement mice, generated as described^53,54^, were bred at the Oregon Health & Sciences University (OHSU) prior to shipment to the Veterans Affairs Puget Sound Health Care System (VAPSHCS). Mice were fed a low-fat (LFD, 10% kcal from fat, Research Diets D12450Bi) or ingredient-matched high-fat diet (HFD, 60% kcal from fat, D12492i) beginning at 9 months of age. Prior to the LFD/HFD, mice had access to a standard chow diet (PicoLab Rodent Diet 20, #5053). Mice had ad libitum access to food and water and were kept on a 12/12 h light/dark cycle. Procedures were completed 6 months later, when the mice were 15 months of age. All procedures complied with the National Institutes of Health Guide for the Care and Use of Laboratory Animals and with IACUC approval at both OHSU and the VAPSHCS. The mice were bred and the study was performed at facilities approved by the Association for Assessment and Accreditation of Laboratory Animal Care International (AAALAC). Sample sizes for each experiment were determined based on expected differences observed in similar previous studies. Within each group, for each experiment, mice were randomly divided. The reporting in this manuscript follows the recommendations in the ARRIVE guidelines.

Each group consisted of 10 mice, with the exception of E3 males on a HFD that consisted of 9 mice. During the 6-month diet period, 5 male E3 HFD, 3 male E4 HFD, and 1 female E3 LFD mice died (Supplementary Fig. 4). All other groups maintained 10 mice each. An additional 4 male E3 LFD and 3 male E3 HFD were added to the study due to the relative larger loss of the male E3 HFD mice. Body weights were collected each month on the diets.

### Radioactive labeling of insulin and albumin

Ten micrograms of human insulin (Sigma-Aldrich, St. Louis, MO, USA) was diluted in 0.25 M chloride-free sodium phosphate buffer (PB), pH 7.5, and radioactivity labeled with 0.5–1 mCi Na^125^I (Perkin Elmer, Waltham, MA, USA) using the chloramine-T (Sigma-Aldrich) method similar to our previously reported methods^40^. Bovine serum albumin (BSA, Sigma-Aldrich) was radioactively labelled with ^99m^Tc (GE Healthcare, Seattle, WA, USA). ^125^I-insulin and ^99m^Tc-albumin were purified on a column of Sephadex G-10 (Sigma-Aldrich). Protein labelling by ^125^I or by ^99m^Tc isotopes was characterized by 15% trichloroacetic acid (TCA) precipitation. Greater than 90% radioactivity in the precipitated fraction was consistently observed for insulin and albumin.

### BBB pharmacokinetic transport of iodinated insulin

Mice were anesthetized with an intraperitoneal injection of 0.1–0.15 ml 40% urethane to minimize pain and distress. Mice received an injection into the right jugular vein of 0.2 ml of 1% BSA/lactated Ringer's solution (LR) containing 1 × 10^6^ cpm of ^125^I-insulin and 5 × 10^5^ cpm of ^99m^Tc-albumin. ^99m^Tc-albumin was co-injected as a marker for vascular space^55^. Blood from the left carotid artery was collected between 0.5 and 10 min after intravenous injection. Mice were immediately decapitated and their whole brains quickly removed and weighed. Each mouse provided a single blood sample and time matched brain sample. The arterial blood was centrifuged at 5400*g* for 10 min at 4 °C and serum collected. The brains were dissected into regions according to the method described in Ref.^56^ and weighed. The levels of radioactivity in serum (50 μl) and brain regions were counted in a gamma counter (Wizard2, Perkin Elmer, Waltham, MA). The brain/serum (B/S) ratios were graphically displayed against their respective exposure times (Expt). Expt was calculated from the formula:

1 $$Exposure\, time= \frac{{\int }_{0}^{t}Cp\left(t\right)dt}{Cp\left(t\right)},$$

where *Cp* is the level of radioactivity (cpm) in serum at time (*t*). Expt corrects for the clearance of peptide from the blood. The influx of insulin was calculated by multiple-time regression analysis as described by Patlaket al.^55,57^:

2 $$\frac{Am}{Cpt}=Ki \left(\frac{{\int }_{0}^{t}Cp\left(t\right)dt}{Cp\left(t\right)}\right)+Vi,$$

where *Am* is level of radioactivity (cpm) per g of brain tissue at time *t*, *Cpt* is the level of radioactivity (cpm) per ml arterial serum at time *t*, *K*_i_ (μl/g-min) is the steady-state rate of unidirectional solute influx from blood to brain. *V*_i_ (μl/g) is the level of rapid and reversible binding for brain which usually is a combination of vascular space plus any brain endothelial cell receptor binding. The brain/serum (B/S) ratios for insulin were corrected for vascular space by subtracting the corresponding ratio for albumin, yielding a delta B/S ratio. The linear portion of the relation between the delta B/S ratio versus Expt was used to calculate the *K*_i_ (μl/g-min) with its standard error term, and the y-intercept determined as representation of the *V*_i_ (μl/g)^55^. As the B/S ratio for ^99m^Tc-albumin did not differ at distinct time points, the vascular space for each genotype and sex was also calculated by collapsing values across time points for each brain region.

### Statistics

As a priori sex differences were expected, male and female data were analyzed separately. Regression analyses and other statistical analyses were performed with Prism 8.0 (GraphPad Software Inc., San Diego, CA, USA) and SPSS (Chicago, IL) software. Brain weight means are reported with their standard error terms and analyzed using two-way analysis of variance (ANOVA) followed by Sidak’s post hoc test when appropriate. For pharmacokinetic studies, the slope of the linear regression lines (*K*_*i*_), reported with their correlation coefficients (*r*), and y-intercepts (*V*_*i*_) were compared statistically using the Prism software, as described^58^.

## Supplementary Information

Supplementary Information.

## References

[1] Freiherr J (2013). Intranasal insulin as a treatment for Alzheimer's disease: A review of basic research and clinical evidence. CNS Drugs.

[2] Craft S (1998). Cerebrospinal fluid and plasma insulin levels in Alzheimer's disease: Relationship to severity of dementia and apolipoprotein E genotype. Neurology.

[3] Ghasemi R (2013). Brain insulin dysregulation: Implication for neurological and neuropsychiatric disorders. Mol. Neurobiol..

[4] Rivera EJ (2005). Insulin and insulin-like growth factor expression and function deteriorate with progression of Alzheimer's disease: Link to brain reductions in acetylcholine. J. Alzheimers Dis..

[5] Liu Y, Liu F, Grundke-Iqbal I, Iqbal K, Gong CX (2011). Deficient brain insulin signalling pathway in Alzheimer's disease and diabetes. J. Pathol..

[6] Steen E (2005). Impaired insulin and insulin-like growth factor expression and signaling mechanisms in Alzheimer's disease—Is this type 3 diabetes?. J. Alzheimers Dis..

[7] Rickle A (2004). Akt activity in Alzheimer's disease and other neurodegenerative disorders. NeuroReport.

[8] Talbot K (2012). Demonstrated brain insulin resistance in Alzheimer's disease patients is associated with IGF-1 resistance, IRS-1 dysregulation, and cognitive decline. J. Clin. Investig..

[9] Banks WA, Jaspan JB, Huang W, Kastin AJ (1997). Transport of insulin across the blood–brain barrier: Saturability at euglycemic doses of insulin. Peptides.

[10] Woods SC, Porte D (1977). Relationship between plasma and cerebrospinal fluid insulin levels of dogs. Am. J. Physiol..

[11] Margolis RU, Altszuler N (1967). Insulin in the cerebrospinal fluid. Nature.

[12] Kaiyala KJ, Prigeon RL, Kahn SE, Woods SC, Schwartz MW (2000). Obesity induced by a high-fat diet is associated with reduced brain insulin transport in dogs. Diabetes.

[13] Heni M (2014). Evidence for altered transport of insulin across the blood–brain barrier in insulin-resistant humans. Acta Diabetol..

[14] Baskin DG (1985). Genetically obese Zucker rats have abnormally low brain insulin content. Life Sci..

[15] Kern W (2006). Low cerebrospinal fluid insulin levels in obese humans. Diabetologia.

[16] Whitmer RA, Gunderson EP, Barrett-Connor E, Quesenberry CP, Yaffe K (2005). Obesity in middle age and future risk of dementia: A 27 year longitudinal population based study. BMJ (Clinical research ed.).

[17] Walker JM, Harrison FE (2015). Shared neuropathological characteristics of obesity, type 2 diabetes and Alzheimer's disease: Impacts on cognitive decline. Nutrients.

[18] Stranahan AM (2008). Diet-induced insulin resistance impairs hippocampal synaptic plasticity and cognition in middle-aged rats. Hippocampus.

[19] Zuloaga KL (2015). High fat diet-induced diabetes in mice exacerbates cognitive deficit due to chronic hypoperfusion. J. Cereb. Blood Flow Metab..

[20] Johnson LA, Torres ER, Impey S, Stevens JF, Raber J (2017). Apolipoprotein E4 and insulin resistance interact to impair cognition and alter the epigenome and metabolome. Sci. Rep..

[21] Pratchayasakul W (2011). Effects of high-fat diet on insulin receptor function in rat hippocampus and the level of neuronal corticosterone. Life Sci..

[22] Bennett DA (2003). Apolipoprotein E epsilon4 allele, AD pathology, and the clinical expression of Alzheimer's disease. Neurology.

[23] Evans DA (1997). Apolipoprotein E epsilon4 and incidence of Alzheimer disease in a community population of older persons. JAMA.

[24] Tanzi RE (2012). The genetics of Alzheimer disease. Cold Spring Harb. Perspect. Med..

[25] Ungar L, Altmann A, Greicius MD (2014). Apolipoprotein E, gender, and Alzheimer's disease: An overlooked, but potent and promising interaction. Brain Imaging Behav..

[26] Harold D (2009). Genome-wide association study identifies variants at CLU and PICALM associated with Alzheimer's disease. Nat. Genet..

[27] Lambert JC (2009). Genome-wide association study identifies variants at CLU and CR1 associated with Alzheimer's disease. Nat. Genet..

[28] Irie F (2008). Enhanced risk for Alzheimer disease in persons with type 2 diabetes and APOE epsilon4: The Cardiovascular Health Study Cognition Study. Arch. Neurol..

[29] Matsuzaki T (2010). Insulin resistance is associated with the pathology of Alzheimer disease: The Hisayama study. Neurology.

[30] Salameh TS, Rhea EM, Banks WA, Hanson AJ (2016). Insulin resistance, dyslipidemia, and apolipoprotein E interactions as mechanisms in cognitive impairment and Alzheimer's disease. Exp. Biol. Med. (Maywood).

[31] Farrer LA (1997). Effects of age, sex, and ethnicity on the association between apolipoprotein E genotype and Alzheimer disease. A meta-analysis. APOE and Alzheimer Disease Meta Analysis Consortium. JAMA.

[32] Fratiglioni L (1997). Very old women at highest risk of dementia and Alzheimer's disease: Incidence data from the Kungsholmen Project, Stockholm. Neurology.

[33] Holland D, Desikan RS, Dale AM, McEvoy LK (2013). Higher rates of decline for women and apolipoprotein E epsilon4 carriers. AJNR Am. J. Neuroradiol..

[34] Lin KA (2015). Marked gender differences in progression of mild cognitive impairment over 8 years. Alzheimer's Dement. Transl. Res. Clin. Interv..

[35] Raber J (1998). Isoform-specific effects of human apolipoprotein E on brain function revealed in ApoE knockout mice: Increased susceptibility of females. Proc. Natl. Acad. Sci. U. S. A..

[36] Raber J, Bongers G, LeFevour A, Buttini M, Mucke L (2002). Androgens protect against apolipoprotein E4-induced cognitive deficits. J. Neurosci..

[37] Raber J (2000). Apolipoprotein E and cognitive performance. Nature.

[38] Hartman RE (2001). Behavioral phenotyping of GFAP-apoE3 and -apoE4 transgenic mice: apoE4 mice show profound working memory impairments in the absence of Alzheimer's-like neuropathology. Exp. Neurol..

[39] Claxton A (2013). Sex and ApoE genotype differences in treatment response to two doses of intranasal insulin in adults with mild cognitive impairment or Alzheimer's disease. J. Alzheimers Dis..

[40] Rhea EM, Torres ERS, Raber J, Banks WA (2020). Insulin BBB pharmacokinetics in young apoE male and female transgenic mice. PLoS One.

[41] Urayama A, Banks WA (2008). Starvation and triglycerides reverse the obesity-induced impairment of insulin transport at the blood–brain barrier. Endocrinology.

[42] Jones NS, Watson KQ, Rebeck GW (2019). Metabolic disturbances of a high-fat diet are dependent on APOE genotype and sex. eNeuro..

[43] Johnson LA (2019). Apolipoprotein E4 mediates insulin resistance-associated cerebrovascular dysfunction and the post-prandial response. J. Cereb. Blood Flow Metab..

[44] Arbones-Mainar JM (2016). Metabolic shifts toward fatty-acid usage and increased thermogenesis are associated with impaired adipogenesis in mice expressing human APOE4. Int. J. Obes. (Lond.).

[45] Jones NS, Rebeck GW (2018). The synergistic effects of APOE genotype and obesity on Alzheimer's disease risk. Int. J. Mol. Sci..

[46] Wolk DA, Dickerson BC, Alzheimer's Disease Neuroimaging, I (2010). Apolipoprotein E (APOE) genotype has dissociable effects on memory and attentional-executive network function in Alzheimer's disease. Proc. Natl. Acad. Sci. U. S. A..

[47] Iadecola C, Gottesman RF (2018). Cerebrovascular alterations in Alzheimer disease. Circ. Res..

[48] Erickson MA, Banks WA (2013). Blood–brain barrier dysfunction as a cause and consequence of Alzheimer's disease. J. Cereb. Blood Flow Metab..

[49] Sweeney MD, Sagare AP, Zlokovic BV (2018). Blood–brain barrier breakdown in Alzheimer disease and other neurodegenerative disorders. Nat. Rev. Neurol..

[50] Banks WA, Reed MJ, Logsdon AF, Rhea EM, Erickson MA (2021). Healthy aging and the blood–brain barrier. Nat. Aging.

[51] Elosua R (2003). Obesity modulates the association among APOE genotype, insulin, and glucose in men. Obes. Res..

[52] Banks WA, Farr SA, Morley JE (2000). Permeability of the blood–brain barrier to albumin and insulin in the young and aged SAMP8 mouse. J. Gerontol. A Biol. Sci. Med. Sci..

[53] Knouff C (1999). Apo E structure determines VLDL clearance and atherosclerosis risk in mice. J. Clin. Investig..

[54] Sullivan PM (1997). Targeted replacement of the mouse apolipoprotein E gene with the common human APOE3 allele enhances diet-induced hypercholesterolemia and atherosclerosis. J. Biol. Chem..

[55] Blasberg RG, Fenstermacher JD, Patlak CS (1983). Transport of alpha-aminoisobutyric acid across brain capillary and cellular membranes. J. Cereb. Blood Flow Metab..

[56] Glowinski J, Iversen LL (1966). Regional studies of catecholamines in the rat brain. I. The disposition of [3H]norepinephrine, [3H]dopamine and [3H]dopa in various regions of the brain. J. Neurochem..

[57] Patlak CS, Blasberg RG, Fenstermacher JD (1983). Graphical evaluation of blood-to-brain transfer constants from multiple-time uptake data. J. Cereb. Blood Flow Metab..

[58] Zar JH (1984). Biostatistical Analysis.

