# Supplementary material for: Effects of apolipoprotein E isoform, sex, and diet on insulin BBB pharmacokinetics in mice
Source: Sci Rep. 2021 Sep 20;11:18636. doi: 10.1038/s41598-021-98061-1 (PMC8452709; doi:10.1038/s41598-021-98061-1)
Supplement: Supplementary file 1 — Supplementary Information. [file 41598_2021_98061_MOESM1_ESM.pdf]

Full Title: Effects of apolipoprotein E isoform, sex, and diet on insulin BBB pharmacokinetics in mice

Short Title: Insulin BBB transport: apoE, sex, and diet

Authors: Elizabeth M. Rhea[1,2*], Kim Hansen[2], Sarah Pemberton[2], Eileen Ruth S Torres[3], Sarah Holden[3], Jacob Raber[3,4], William A. Banks[1,2]

[1] Department of Medicine, Division of Gerontology and Geriatric Medicine, University of Washington, Seattle, WA 98195

[2] Geriatric Research Education and Clinical Center, Veterans Affairs Puget Sound Health Care System, Seattle, WA 98108

[3] Department of Behavioral Neuroscience, Oregon Health & Science University, Portland, Oregon 97239

[4] Departments of Neurology and Radiation Medicine, Division of Neuroscience, ONPRC, Oregon Health & Science University, Portland, Oregon 97239

*Corresponding author: Elizabeth M. Rhea, meredime@uw.edu, 1660 S. Columbian Way, Seattle, WA 98108

Keywords:

Blood-brain barrier, apolipoprotein E, sex, diet, insulin, pharmacokinetics

Supplementary Information

Supplemental Figure 1

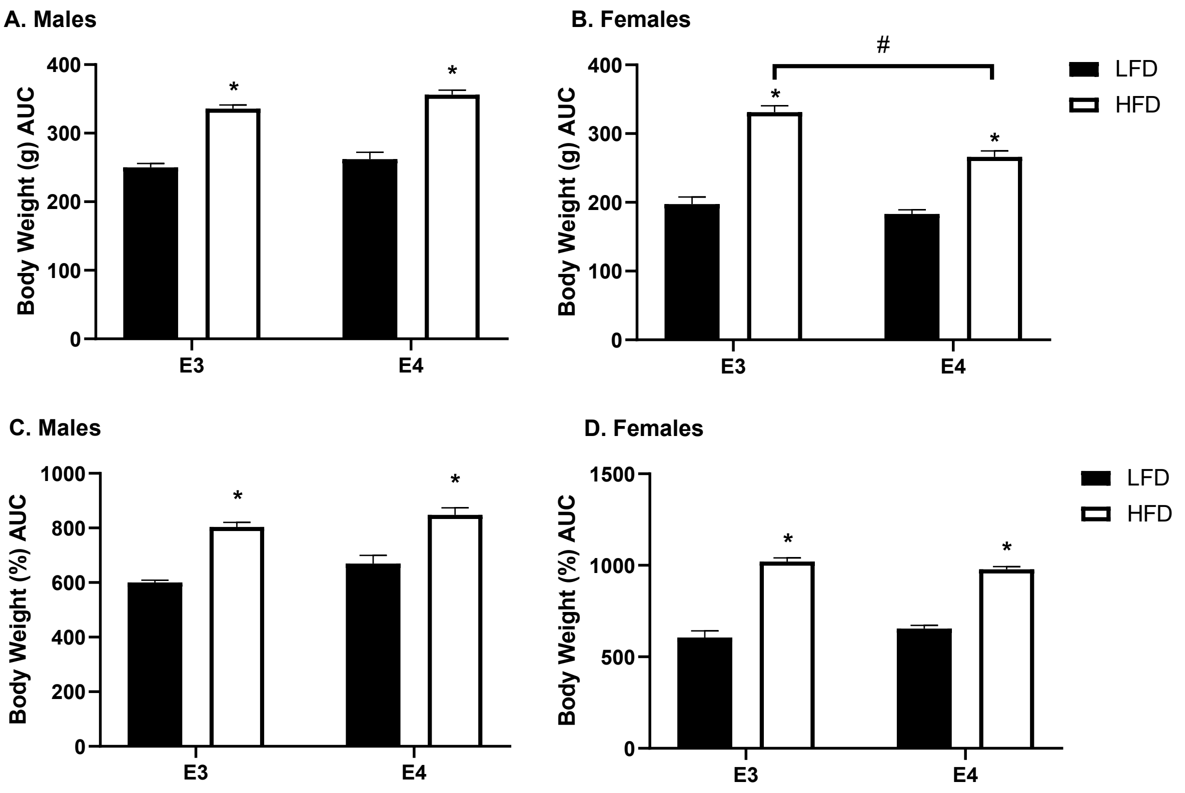

Supplemental Figure 1. Area under the curve (AUC) analysis of the body weight curves presented in Figure 1. A-D represent the AUC for each panel respectively in Figure 1. Two-way ANOVA *p<0.05 vs respective LFD group. #p<0.05 vs genotype within diet.

Supplemental Figure 2

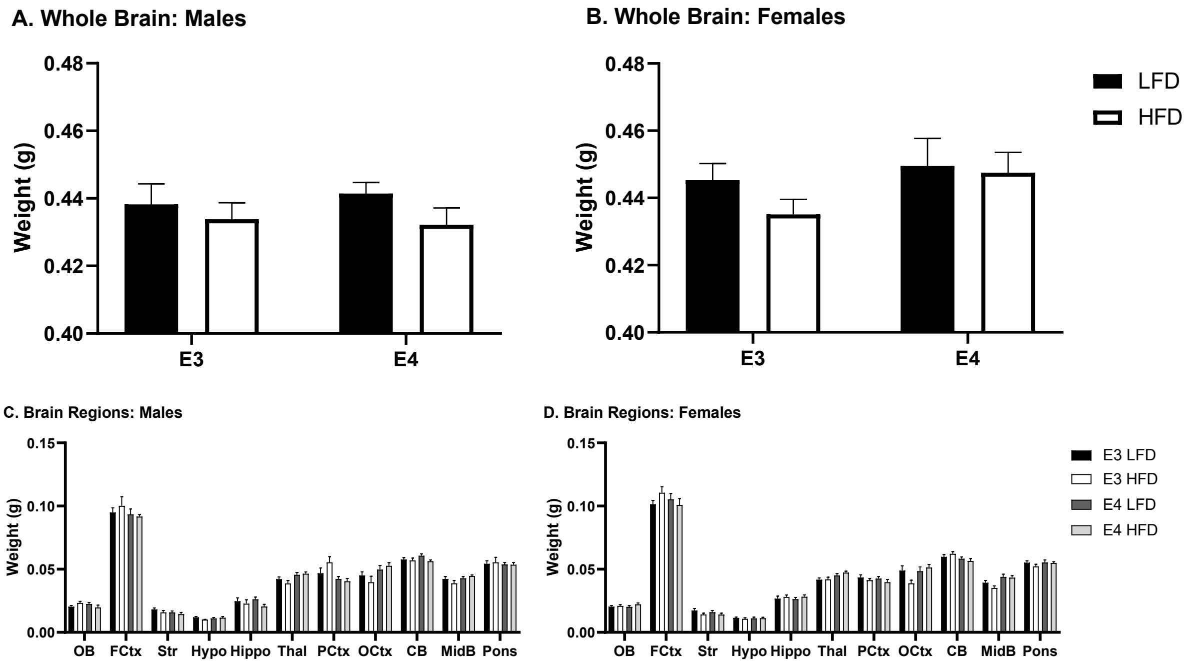

Supplemental Figure 2. Brain weights. Whole brain weights for A) males and B) females were analyzed by a two-way ANOVA and did not statistically differ. Brain region weights for C) males and D) females were analyzed by repeated measures and had significant interactions between the brain region weights and genotype (p<0.02). While there was no significant effect of diet in males or E4 females, E3 females did exhibit significant differences (p<0.02). OB- olfactory bulb, FCtx- frontal cortex, Str- striatum, Hypo- hypothalamus, Hippo- hippocampus, Thal- thalamus, PCtx- parietal cortex, OCtx- occipital cortex, CB- cerebellum, MidB- midbrain, Pons- Pons/medulla

Supplemental Figure 3

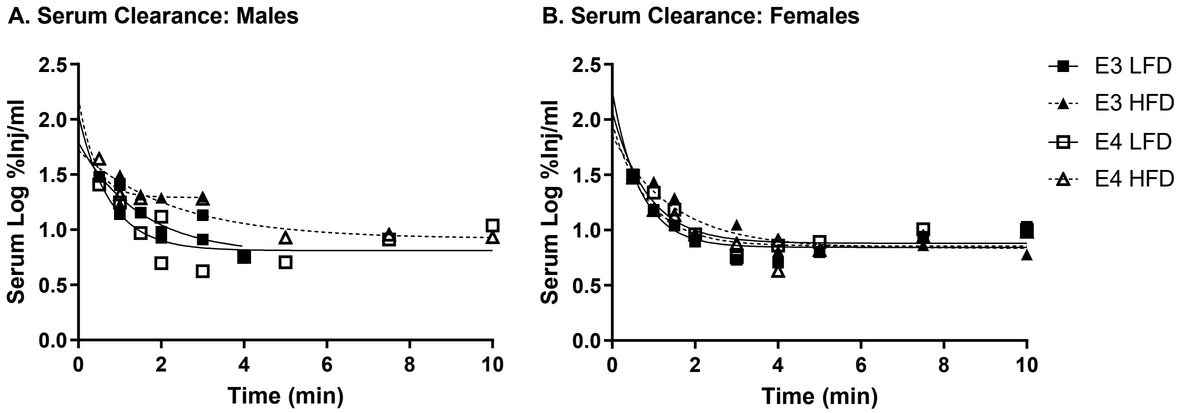

Supplemental Figure 3. Full ^{125}I-insulin serum clearance curves. ^{125}I-Insulin serum clearance in A) males and B) females over time. Serum clearance in males is more variable due to diet than in females. Linear clearance is expressed in Figure 3.

Supplemental Figure 4

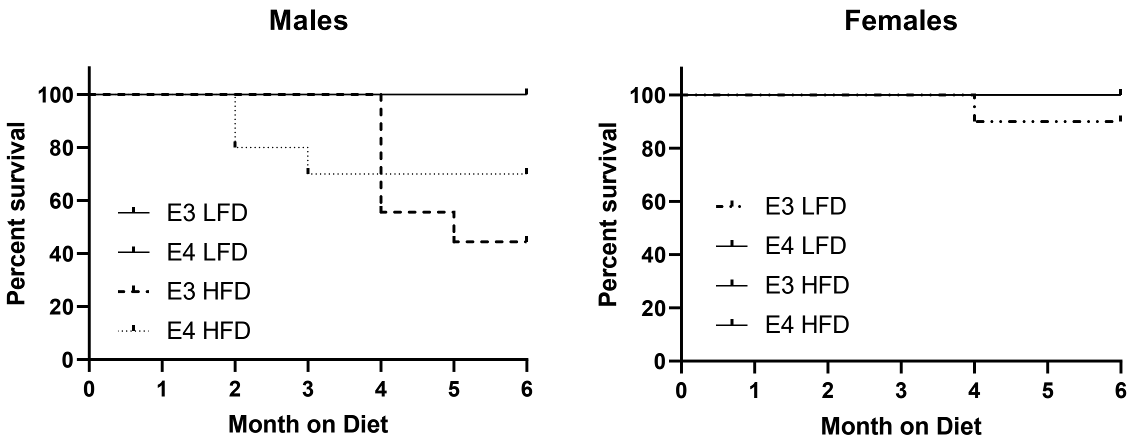

Supplemental Figure 4. Kaplan-Meier survival curves for initial mouse cohorts. Percent survival is expressed for each initial group over the course of the 6-month diet. Log-rank test shows a significant difference for males (p=0.0103) but not females (p=0.3916).

Supplemental Table 1. ^{125}I-insulin regional vascular binding in females.

FEMALES	E3 LFD		E3 HFD		E4 LFD		E4 HFD	
**Region**	V_i	**SEM**	V_i	**SEM**	V_i	**SEM**	V_i	**SEM**
Olfactory Bulb	11.7	6.4	6.6	3.3	12.2	6.0	6.6	2.8
Frontal Cortex	6.3	2.1	2.4	1.4	5.3	1.6	5.2	1.3
Striatum	4.0	2.4	2.2	3.2	6.3	2.9	2.5	3.4
Hypothalamus	-0.2	3.8	6.4	0.9	4.4	3.2	3.8	4.0
Hippocampus	5.1	0.5	5.8	0.3	8.8	0.6	3.7	0.5
Thalamus	8.4	4.6	4.9	2.6	8.8	3.9	6.4	2.9
Parietal Cortex	6.1	3.9	2.3	1.0	7.9	2.3	3.7	0.7
Occipital Cortex	4.7	2.7	3.3	1.3	3.9	2.7	4.1	2.1
Cerebellum	11.4	5.6	6.1	4.0	10.7	5.3	11.4	3.9
Midbrain	3.2	2.8	2.1	1.8	5.4	1.7	1.5	1.4
Pons/Medulla	7.9	1.9	4.4	2.1	9.4	2.8	3.0	1.5

There is no statistical difference in the level of ^{125}I-insulin vascular binding. V_i: reversible vascular binding, SEM: ± standard error mean.

Supplemental Table 2. ^{125}I-insulin regional BBB transport in males.

**MALES**	**E3 LFD**				**E3 HFD**				**E4 LFD**				**E4 HFD**			
**Region**	K_i	**SEM**	**r**	***p***	K_i	**SEM**	**r**	***p***	K_i	**SEM**	**r**	***p***	K_i	**SEM**	**r**	***p***
Olfactory Bulb	1.28	0.38	0.69	0.006	1.90	0.45	0.90	0.014	0.99	0.35	0.73	0.027	1.07	0.33	0.85	0.030
Frontal Cortex	0.69	0.12	0.85	0.000	0.31	0.32	0.44	0.382	0.65	0.16	0.84	0.004	0.32	0.07	0.89	0.007
Striatum	0.59	0.29	0.51	0.062	0.04	0.37	0.06	0.912	0.57	0.33	0.52	0.127	-0.12	0.34	0.15	0.740
Hypothalamus	1.22	0.40	0.66	0.010	0.57	0.75	0.36	0.487	0.46	0.29	0.49	0.155	0.35	0.33	0.43	0.337
Hippocampus	0.92	0.36	0.59	0.027	1.08	0.80	0.56	0.252	1.28	0.45	0.71	0.022	0.36	0.17	0.69	0.086
Thalamus	0.56	0.27	0.51	0.061	0.65	0.26	0.79	0.064	0.89	0.39	0.65	0.057	0.34	0.15	0.71	0.076
Parietal Cortex	0.58	0.17	0.70	0.005	-0.07	0.28	0.12	0.814	0.73	0.27	0.69	0.026	0.45	0.12	0.86	0.014
Occipital Cortex	0.75	0.23	0.69	0.006	0.88	0.31	0.81	0.049	0.79	0.21	0.80	0.006	0.48	0.14	0.83	0.020
Cerebellum	1.06	0.29	0.72	0.004	1.08	0.33	0.85	0.032	0.99	0.30	0.78	0.014	0.44	0.13	0.83	0.021
Midbrain	0.40	0.15	0.60	0.023	0.96	0.43	0.74	0.090	0.53	0.18	0.75	0.020	0.22	0.05	0.87	0.011
Pons/Medulla	1.41	0.40	0.71	0.004	0.76	0.26	0.82	0.045	1.07	0.39	0.72	0.028	0.65	0.18	0.87	0.024

Black cells represent regions where there was no significant ^{125}I-insulin BBB transport (*$p > 0.05$ linear regression). K_i: linear transport, SEM: ± standard error mean, r: correlation coefficient, *p:* significance for linear regression

Supplemental Table 3. ^{125}I-insulin regional BBB transport in females.

FEMALES	E3 LFD				E3 HFD				E4 LFD				E4 HFD			
Region	Ki	SEM	r	p	Ki	SEM	r	p	Ki	SEM	r	p	Ki	SEM	r	p
Olfactory Bulb	1.06	0.53	0.60	0.087	1.19	0.34	0.80	0.010	0.99	0.60	0.50	0.140	1.34	0.30	0.86	0.003
Frontal Cortex	0.49	0.17	0.73	0.025	1.01	0.14	0.94	0.000	0.76	0.16	0.85	0.002	0.62	0.14	0.86	0.003
Striatum	0.56	0.20	0.73	0.027	1.32	0.33	0.83	0.005	0.30	0.30	0.33	0.346	0.85	0.37	0.65	0.057
Hypothalamus	1.40	0.31	0.86	0.003	0.89	0.10	0.96	0.000	1.12	0.32	0.77	0.009	1.41	0.43	0.77	0.014
Hippocampus	1.43	0.54	0.71	0.034	0.96	0.30	0.79	0.020	1.07	0.59	0.54	0.104	1.23	0.45	0.72	0.030
Thalamus	0.71	0.38	0.57	0.107	0.91	0.27	0.79	0.011	0.74	0.40	0.57	0.105	0.99	0.31	0.77	0.016
Parietal Cortex	0.64	0.33	0.60	0.090	0.95	0.10	0.96	<0.0001	0.48	0.23	0.60	0.070	0.92	0.08	0.98	<0.0001
Occipital Cortex	0.73	0.23	0.77	0.015	1.03	0.14	0.95	0.000	1.01	0.26	0.83	0.006	1.01	0.23	0.85	0.004
Cerebellum	1.17	0.47	0.69	0.042	1.34	0.41	0.77	0.014	1.63	0.54	0.73	0.017	1.04	0.43	0.67	0.047
Midbrain	0.78	0.23	0.79	0.012	0.93	0.19	0.89	0.002	0.50	0.18	0.71	0.022	0.82	0.15	0.90	0.001
Pons/Medulla	0.91	0.16	0.90	0.001	1.31	0.22	0.92	0.001	0.81	0.28	0.71	0.021	1.27	0.16	0.94	<0.0001

Black cells represent regions where there was no significant ^{125}I-insulin BBB transport (*p > 0.05 linear regression). K_i: linear transport, SEM: ± standard error mean, r: correlation coefficient, *p:* significance for linear regression
